# FOXO4 peptide targets myofibroblast ameliorates bleomycin‐induced pulmonary fibrosis in mice through ECM‐receptor interaction pathway

**DOI:** 10.1111/jcmm.17333

**Published:** 2022-05-05

**Authors:** Xiaodan Han, Tong Yuan, Junling Zhang, Yonggang Shi, Deguan Li, Yinping Dong, Saijun Fan

**Affiliations:** ^1^ Tianjin Key Laboratory of Radiation Medicine and Molecular Nuclear Medicine Institute of Radiation Medicine Peking Union Medical College and Chinese Academy of Medical Science Tianjin China; ^2^ Department of Radiation Oncology the First Affiliated Hospital of Zhengzhou University Zhengzhou China

**Keywords:** bleomycin, FOXO4‐DRI, myofibroblast, pulmonary fibrosis

## Abstract

Pulmonary fibrosis (PF) is a progressive interstitial lung disease with limited treatment options. The incidence and prevalence of PF is increasing with age, cell senescence has been proposed as a pathogenic driver, the clearance of senescent cells could improve lung function in PF. FOXO4‐D‐Retro‐Inverso (FOXO4‐DRI), a synthesis peptide, has been reported to selectively kill senescent cells in aged mice. However, it remains unknown if FOXO4‐DRI could clear senescent cells in PF and reverse this disease. In this study, we explored the effect of FOXO4‐DRI on bleomycin (BLM)‐induced PF mouse model. We found that similar as the approved medication Pirfenidone, FOXO4‐DRI decreased senescent cells, downregulated the expression of senescence‐associated secretory phenotype (SASP) and attenuated BLM‐induced morphological changes and collagen deposition. Furthermore, FOXO4‐DRI could increase the percentage of type 2 alveolar epithelial cells (AEC2) and fibroblasts, and decrease the myofibroblasts in bleomycin (BLM)‐induced PF mouse model. Compared with mouse and human lung fibroblast cell lines, FOXO4‐DRI is inclined to kill TGF‐β‐induced myofibroblast in vitro. The inhibited effect of FOXO4‐DRI on myofibroblast lead to a downregulation of extracellular matrix (ECM) receptor interaction pathway in BLM‐induced PF. Above all, FOXO4‐DRI ameliorates BLM‐induced PF in mouse and may be served as a viable therapeutic option for PF.

## 1 INTRODUCTION

Pulmonary fibrosis (PF) is a progressive interstitial lung disease with a median survival of 3–5 years.^1^ Although the pathogenesis of PF is not fully understood, some researches have suggested that repetitive micro‐injuries to the alveolar epithelium initiate the histological changes in PF.^2^ These micro‐injuries lead to the initial damage and aseptic inflammation of alveolar epithelial‐mesenchymal unit and will not be properly repaired. Continuous disruption of the alveolar epithelial cell (AEC) layer promotes destruction of the basal membrane and the subsequent activation of an intra‐alveolar coagulation cascade, including imbalance of matrix metalloproteinases (MMPs) and tissue inhibitors of metalloproteinases (TIMPs), activation of myofibroblasts, ultimately results in the excessive deposition of extracellular matrix.^2^

The causes of PF are concerned with age, genomic instability, environmental factors, radiation exposure, smoking, viral infections and so on.^2^, ^3^ In fact, one of the main prognostic factors of PF in epidemiological studies is age.^3^, ^4^ An increasing body of evidence has suggested that there is a link between ageing(include cell senescence) and PF pathogenesis.^4^, ^5^, ^6^ The established biomarkers of cell senescence, including p16^Ink4a^, p21 and β‐galactosidase (β‐gal) have been observed in human PF lung tissue.^7^, ^8^ The pro‐ageing stressor is implicated in PF pathogenesis, including DNA damage, oxidative stress, telomere attrition and proteome instability.^3^, ^6^ Importantly, elimination of senescent cells has been reported to restore lung structure, compliance and elasticity in aged mice.^9^

Pirfenidone (PFD) is listed as one of only two novel agents conditionally recommended for the treatment of PF, it could attenuate lung fibrosis by suppression of transforming growth factor (TGF‐β) and other growth factors. However, treatment‐emergent adverse events including upper respiratory infection, bronchitis, gastrointestinal events (nausea and diarrhoea), fatigue, cough and dyspnoea sometimes limit its application.^10^ The findings of previous researches on cell senescence and PF suggest that senescence clearance plays a crucial role in PF pathogenesis and improves lung function.^9^ FOXO4‐D‐Retro‐Inverso (FOXO4‐DRI), a peptide to perturb FOXO4 interaction with p53, has been found to selectively induce apoptosis of senescent cells.^11^ More importantly, even the loss of health has occurred, FOXO4‐DRI effectively restores tissue homeostasis in aged mice.^11^ In this study, we examined the effect of FOXO4‐DRI on PF using a bleomycin (BLM)‐induced PF mouse model.

## 2 MATERIALS AND METHODS

### 2.1 Mice

Male C57BL/6J mice were purchased from Beijing Huafukang Bioscience Co. Inc. Mice were used at approximately 6–8 weeks of age. All use of animals in this study were approved by the Animal Care and Ethics Committee of the Institute of Radiation Medicine, Peking Union Medical College and Chinese Academy of Medical Sciences. All experimental procedures in this study were complied with the Guide for the Care and Use of Laboratory Animals and the National Institutes of Health guide for the Care and Use of Laboratory Animals.

### 2.2 FOXO4‐DRI development

FOXO4‐DRI was synthesized as previously described.^11^ It was manufactured at >95% purity and stored at −20℃ in 1mg powder aliquots to avoid freeze‐thawing artefacts. For in vivo experiment, FOXO4‐DRI was dissolved in sterile PBS to generate a 5 mg/ml stock solution. Before intraperitoneal injection, FOXO4‐DRI stock solution was diluted in PBS to a concentration of 1 mg/ml.

### 2.3 BLM‐induced PF model and FOXO4‐DRI administration

BLM (Solarbio) was diluted in sterile PBS to a concentration of 1 mg/ml. Under 3.5% chloral hydrate anaesthesia, mice were administrated with 5 mg/kg BLM or with 100 μl PBS by intratracheal instillation. Mice were administrated with 5 mg/kg FOXO4‐DRI or PBS by intraperitoneal injection on the 14^th^, 16^th^ and 18^th^ day after BLM administration. For positive control, mice were administrated with 300 mg/kg PFD from the 1^st^ day after BLM administration for 20 times. Twenty‐one days after BLM administration, these mice were euthanized.

### 2.4 Histopathologic evaluation and IHC

Fresh lung tissue was harvested and fixed with 4% formalin, embedded with paraffin, serially sectioned and stained with haematoxylin and eosin. To evaluate collagen deposition, the paraffin‐embedded lung tissue was stained using a Masson trichrome kit (Solarbio), according to the manufacturer's instructions. Collagen deposition was qualified by Image Pro Plus software (Media Cybernetics) on Masson trichrome‐stained sections. The data are illustrated as ratio of positive (blue) area relative to the total lung area under 10 microscopic fields as described previously.^12^ For the IHC experiments, the primary antibodies of α‐SMA and Col1a1(Bioworld) were incubated overnight at 4°C, after secondary antibody conjunction, a DAB kit was used to detect the positive staining.

### 2.5 Measurement of hydroxyproline content

Frozen lung tissue was homogenized in water. Hydroxyproline content in the homogenate was measured by a hydroxyproline colorimetric assay kit (Bio Vision), according to the manufacturer's instructions.

### 2.6 β‐galactosidase (β‐gal) staining

Frozen lung sections were stained with a β‐gal staining kit (Beyotime Biotechnology) according to the manufacturer's instructions. Senescent cells were identified as blue‐stained under optical microscopy.

### 2.7 Quantitative real‐time PCR

Total RNA was extracted from lung tissue with TRIzol (Life Technologies). cDNA synthesis was carried out with a Revert Aid First Strand cDNA Synthesis Kit (Thermo Scientific), according to the manufacturer's protocol. Quantitative real‐time PCR was performed using SYBR Green Master (Roche) under an ABI 7500 Sequence Detection System (Thermo). The primer sequences were listed in Table 1.

**TABLE 1:** Primer sequences used in experiments

Gene	Forward	Reverse
TGF‐β1	CAATTCCTGGCGTTACCT	CTGTATTCCGTCTCCTTGG
Col1α2	GCGATTACTACTGGATTGAC	GCGGCTGTATGAGTTCTT
Col3α1	TGGTGCTAAGGGTGAAGT	CCAGGACTGCCGTTATTC
Timp1	CTTGGTTCCCTGGCGTACTC	ACCTGATCCGTCCACAAACAG
Mmp2	AAGATTGACGCTGTGTATGA	CATCTACTTGCTGGACATCA
p21	CCTGGTGATGTCCGACCTG	CCATGAGCGCATCGCAATC
p16	CGCAGGTTCTTGGTCACTGT	TGTTCACGAAAGCCAGAGCG
Tnf‐α	TGAAAACGGAGCTGAGCTGT	CTCTCAATGACCCGTAGGGC
Pai1	TCCACAAGTCTGATGGCAGC	TGGTAGGGCAGTTCCACAAC
Mmp10	AAGACCTGAGACCCCAGACA	GTGAGCCTCATAGGCAGCAT
Mmp13	TTCACTGCGAGCGTTCAGAT	AGCCAAATGTAAGGCCACCT
IL1a	AGGGAGTCAACTCATTGGCG	TGGCAGAACTGTAGTCTTCGT
IL1b	TGCCACCTTTTGACAGTGATG	TGATGTGCTGCTGCGAGATT
Fn1	CTCCATTCCACCTTACAACA	TCAAGCCAGACACAACAAT
Lama1	ACTATGCCGTCAGCGATACAG	GGCACCAGCTTTGAATAATACGA
Tnc	GCTCTCCTATGGCATCAAG	TACTCCGTGTCAGGTCTC
Hmmr	ACGGCTTACTGAATTAACCA	GACCATCATACTCCTCATCTT
Thbs2	GCTACTAATGCCACCTACC	TCCTTCTCATCGCTCACA
Cytl1	CTGCTACTCTCGGATGCT	AGGAGAAGCCACGAAGTC
Gc	AATTGGCAGAACGGCTAA	TTATAGAGCAGCACTTAGAGG
Nrgn	GACGACGATATTCTTGACATC	TCCGCTCTTTATCTTCTTCC
Sele	GGATAACGAGACGCCATC	GTCCGTCTCAGAAGAATAGG
Dpysl5	GTCAATGTGTCTAGTATCTCAG	GAGGTAGGTGGAAGTGTTG
Chil4	AAGACTTGCGTGACTATGAA	CGAAGGAATCTGATAACTGAC
Gpr176	TGTCACCAACAGGTTCATTA	GCAGAAGAGCATCGTATAGA
Krt6a	GGACAGCATCATTGGAGAG	GCAGCATCTACATCCTTCTT
Mex3a	CAAGTGACCATCCGTGTA	CCGTCGCTGTTGTATTCA
col4a2	CCAGGTTTTAAAGGCAGCCG	TTTGCGCCCAGGTATCCTTT
Ntrk2	AAGTTGGCGAGACATTCC	CCGAAGAAGATGGAGTGTT
Pappa2	ACCATTGCTACCTTCGCATATC	GGAAACCTTGAGGGGTATTCTG
Tgm5	AGTGATGAGCGTGATGAC	TGAGACCTTGTAGCCTGTA
Myh2	TAAACGCAAGTGCCATTCCTG	GGGTCCGGGTAATAAGCTGG
β‐tubulin	GGATGATGCGCTTGTTCGC	AGTTCATCATCCACTCAAGGTGT

### 2.8 Western blot

Lung tissues were homogenized in liquid nitrogen and then lysed in RIPA buffer (Solarbio) plus phenylmethylsulfonyl fluoride (Sigma) on ice. The protein samples were subjected to 10% or 12% SDS‐PAGE, transferred to PVDF membranes (Millipore) and incubated with an antibody against β‐tubulin (1:1000), p16^Ink4a^ (1:1000), p21 (1:1000), Tenascin (1:500), Thbs2 (1:500), Laminin1 (1:1000), Fibronectin (1:1000), Hmmr (1:500), α‐SMA (1:1000), Col1a1(1:1000) and FOXO4(1:500).

### 2.9 RNA‐Seq

Total RNA was extracted from lung tissues, RNA integrity was evaluated and then the libraries were constructed. These libraries were sequenced on the Illumina sequencing platform (HiSeqTM 2500 or Illumina HiSeq X Ten) and 125 bp/150 bp paired‐end reads were generated. Raw reads were processed and analysed with the help of OEbiotech Company.

### 2.10 Flow cytometry

The lung cell suspension was prepared as previously described.^6^ In briefly, mouse lung was harvested, washed with PBS to remove blood, then cut into pieces, which incubated in the DMEM medium containing 0.2 mg/ml Liberase DL (Roche) and 0.03 mg/ml DNase I (Roche) at 37℃ for 40 min. 5 × 10^6^ cells were stained with the following antibodies (Biolegend), CD45‐BV510, CD31‐APC, PDGFa‐PE, EPCAM ‐Percp, PDPN‐BV421 and MHCII‐APC‐Cy7 at 4℃ for 30 min. According to previously researches,^6^, ^13^ CD45^−^CD31^+^EPCAM^−^ cells were named as endothelial cells, further marked with PDPN to distinguish vascular endothelial cells(PDPN^−^) and lymphocytic endothelial cells(PDPN^+^); CD45^−^CD31^−^EPCAM^+^ cells were named as AECs, further sorted into AEC1(PDPN^+^) and AEC2(MHCⅡ^+^); Fibroblasts were marked as CD45^−^PDGFα^+^EPCAM^−^ cells. For intracellular staining, the lung cells were fixed and permeabilized using BD Cytofix/Cytoperm buffer first, α‐SMA and h‐caldesmon antibodies were used to detected myofibroblasts and smooth muscle cells, the mean fluorescence intensity (MFI) of α‐SMA and h‐caldesmon was detected by a BD Aria Ⅲ flow cytometer (BD Bioscience).

### 2.11 Cell lines, mouse fibroblast isolation and culture

Human lung fibroblast (HLF and MRC5) was purchased from National Collection of Authenticated Cell Cultures (Shanghai, China). For the mouse lung fibroblast (MLF) culture, the lung cells were isolated first as described in the above section, then all the cells were cultured in DMEM plus 20% FBS for 24 h, after suspension cells were removed, the adherent cells could be considered as MLF. Passage 3–6 MLF were used for the remaining experiments. To detect IC50 value, cells were treated with 4 ng/ml TGF‐β for 24 h, then co‐cultured with a series dilution of FOXO‐DRI for 24, 48 and 72 h; CCK‐8 assay was executed and the OD value was detected. For apoptosis, 1 × 10^5^ cells were treated as described above, then detected according to the manufacturer's instructions (BD Bioscience).

### 2.12 Statistical analysis

Statistical analyses were performed using GraphPad Prism 8 software. Significant differences between groups were evaluated by One‐way analysis of variance, followed by a post‐hoc student‐Newman–Keuls test for multiple comparisons. Differences were considered significant at *p *< 0.05.

## 3 RESULTS

### 3.1 FOXO4‐DRI eliminates cell senescence and ameliorates BLM‐induced PF

**FIGURE 1:**
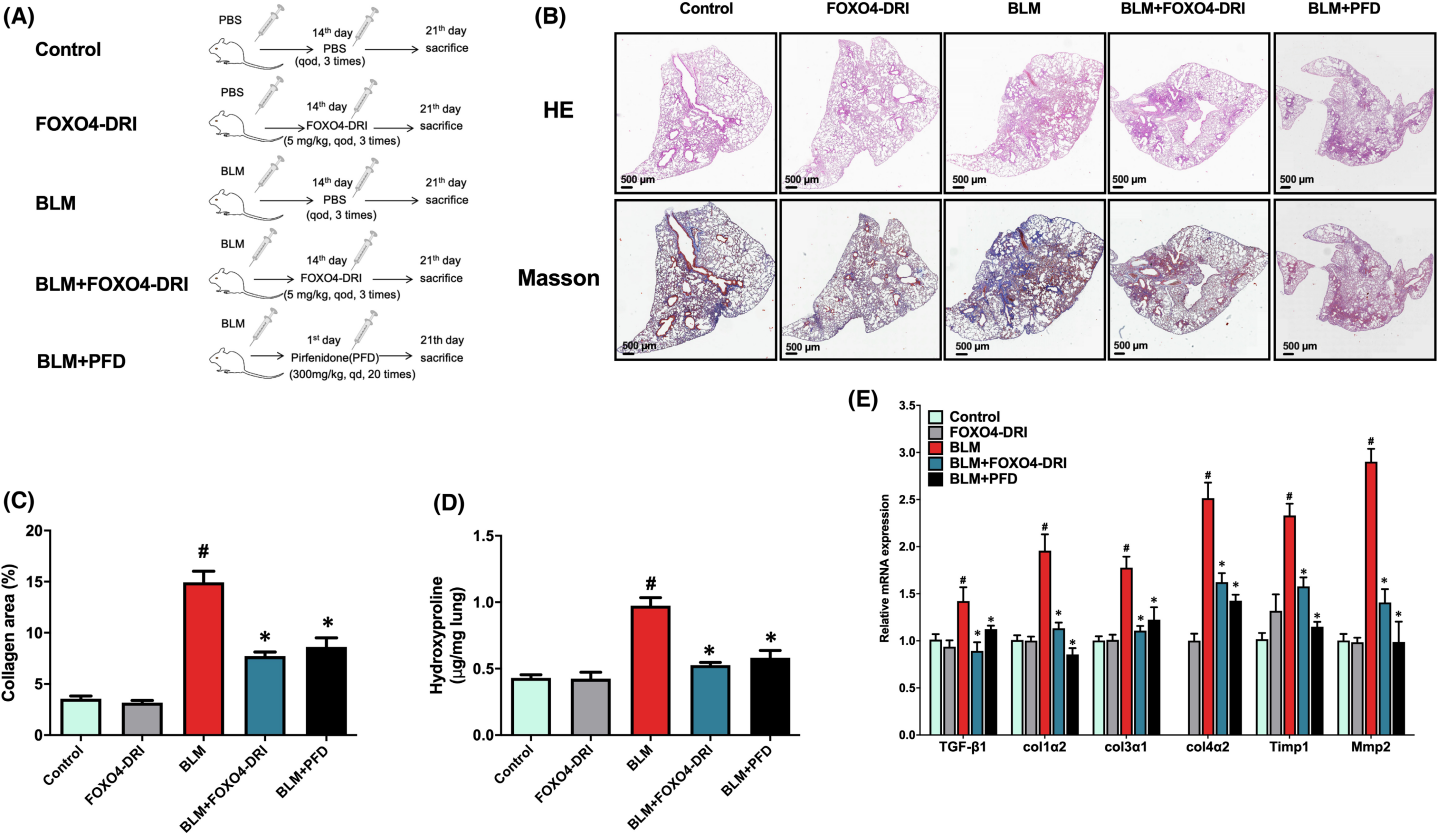
FOXO4‐D‐Retro‐Inverso(FOXO4‐DRI) ameliorates bleomycin (BLM)‐induced pulmonary fibrosis (PF). (A) Schematic diagram illustrating the experimental design. (B) Representative scanned images of haematoxylin‐eosin (HE) staining and Masson trichrome‐staining of lung tissue sections. Collagen deposition was qualified by Masson trichrome‐staining and the measurement of hydroxyproline content, and the data were expressed as (C) the percentage of collagen deposited areas relative to total lung tissue areas and (D) hydroxyproline content in 1 mg lung tissue. (E) The relative expression of TGF‐β1, Col1α2, Col3α1, Col4α2, Timp1 and Mmp2 mRNA in lung tissues. All the data were represented as mean ± SEM (*n* = 3 in panel C and E); *n* = 5 in panel (B). ^#^ *p *< 0.05 vs control; *p < 0.05 vs BLM

It has been well established that BLM aerosol or intratracheal instillation in mice can lead to PF that recapitulates key features of human PF.^14^ In mouse, lung fibrosis appears between 14 and 28 days after a single‐dose administration of bleomycin.^2^, ^15^ Therefore, 14 days after the mice were intratracheal instilled with BLM, they were treated with PBS or FOXO4‐DRI by intraperitoneal injection for 3 times, and the positive control PFD by oral gavage for 20 times (Figure 1A). Twenty‐one days after BLM administration, these mice were euthanized. Senescent cells have been found in the lung with PF, and it has been established that fibrotic lung disease is mediated, in part, by senescent cells.^6^ FOXO4‐DRI selectively induce senescent cells apoptosis, restores fur density, fitness and renal function in aged mice.^11^ Therefore, we first examined whether FOXO4‐DRI could clear senescent cells in BLM ‐induced PF model. As shown in Figure S1A,B, the number of β‐gal positive cells was significantly higher than that in control mice at 21^th^ days after BLM administration; FOXO4‐DRI treatment substantially reduced the BLM‐induced β‐gal positive cells, suggesting that BLM causes cell senescence and FOXO4‐DRI eliminates senescent cells in the PF model. Senescent cells can develop a senescence‐associated secretory phenotype (SASP), which include interleukins, proteases and regulators, chemokines, insoluble factors and so on.^16^ SASP can exert profound effects on themselves or on neighbouring cells.^17^ As shown in Figure S1C–E, there was significant upregulated expression of PF‐related SASP after mice were treated with BLM, and FOXO4‐DRI can downregulated these genes expression. p16^Ink4a^ and p21 are the main senescence hallmarks, the results of quantitative real‐time PCR and western blot showed that mouse in BLM group exhibited upregulation of p16^Ink4a^ and p21 expression in lung compared with control, FOXO4‐DRI significantly downregulated p16^Ink4a^ and p21 expression in the lung of mice with BLM‐induced PF (Figure S1E–G). These results suggest FOXO4‐DRI clears senescent cells in the PF model and regulates senescence‐associated genes.

The strategy of senescent cells clearance had been considered as a nice treatment for PF, so next we explored the effect of FOXO4‐DRI on BLM‐induced mouse PF model. HE staining revealed that the lungs in control and FOXO4‐DRI groups maintained normal histological morphology, which meant normal alveoli and alveolar septum, no abnormal inflammatory cell infiltration and collagen deposition. BLM induced a widening alveolar septum, inflammatory cell infiltration and collagen deposition in the lung, FOXO4‐DRI significantly ameliorated BLM‐induced morphological changes to positive control level in the lung (Figure 1B). The lung sections were also stained using a Masson trichrome kit to evaluate collagen deposition. As shown in Figure 1B,C, compared with control mice, the collagen deposition (blue‐stained areas) in the lung was significantly increased in BLM group, and FOXO4‐DRI reduced BLM‐induced lung collagen deposition similar to PFD group. Meanwhile, mice in BLM group showed a significantly higher hydroxyproline content in the lung, and FOXO4‐DRI decreased the level of hydroxyproline content (Figure 1D).

We next explored the mRNA level of fibrosis relevant genes in lung, a distinct feature of PF is collagen deposition with the upregulation of collagen genes, such as Col1α2, Col3α1and Col4a2.^18^ BLM induced the upregulation of Col1α2, Col3α1 and Col4a2 mRNA expression compared with control, and FOXO4‐DRI repressed BLM‐induced upregulation of the above genes similar to PFD group (Figure 1E). Apart from collagen deposition, the dysregulation of collagen degradation also contributes to the development of PF. Collagen degradation is mainly performed by matrix metalloproteinases(MMP) family, it has been reported that Mmp2, a major type IV collagen‐degradation enzyme, and its inhibitor Timp1 is increased in the lung tissues of BLM‐treated mice.^19^, ^20^ In our experiment, BLM induced a remarkable upregulation of Timp1 and Mmp2 mRNA and FOXO4‐DRI downregulated their expression similar to PFD group (Figure 1E). These results suggest that FOXO4‐DRI represses collagen deposition to ameliorate BLM‐induced PF. TGF‐β is mainly contribute for the differentiation of fibroblasts into myofibroblasts and mesenchymal transition of epithelial cells.^21^, ^22^, ^23^ In our experiment, BLM upregulated the expression of TGF‐β1 mRNA level, and FOXO4‐DRI downregulated TGF‐β1 expression in BLM treatment mouse (Figure 1E).

In summary, above data suggests that intratracheal instillation of BLM induces PF in mice, and FOXO4‐DRI, similar as positive control PFD, attenuates BLM‐induced pathological changes in the lung.

### 3.2 FOXO4‐DRI improves the impaired ratio in BLM‐induced lung cells

**FIGURE 2:**
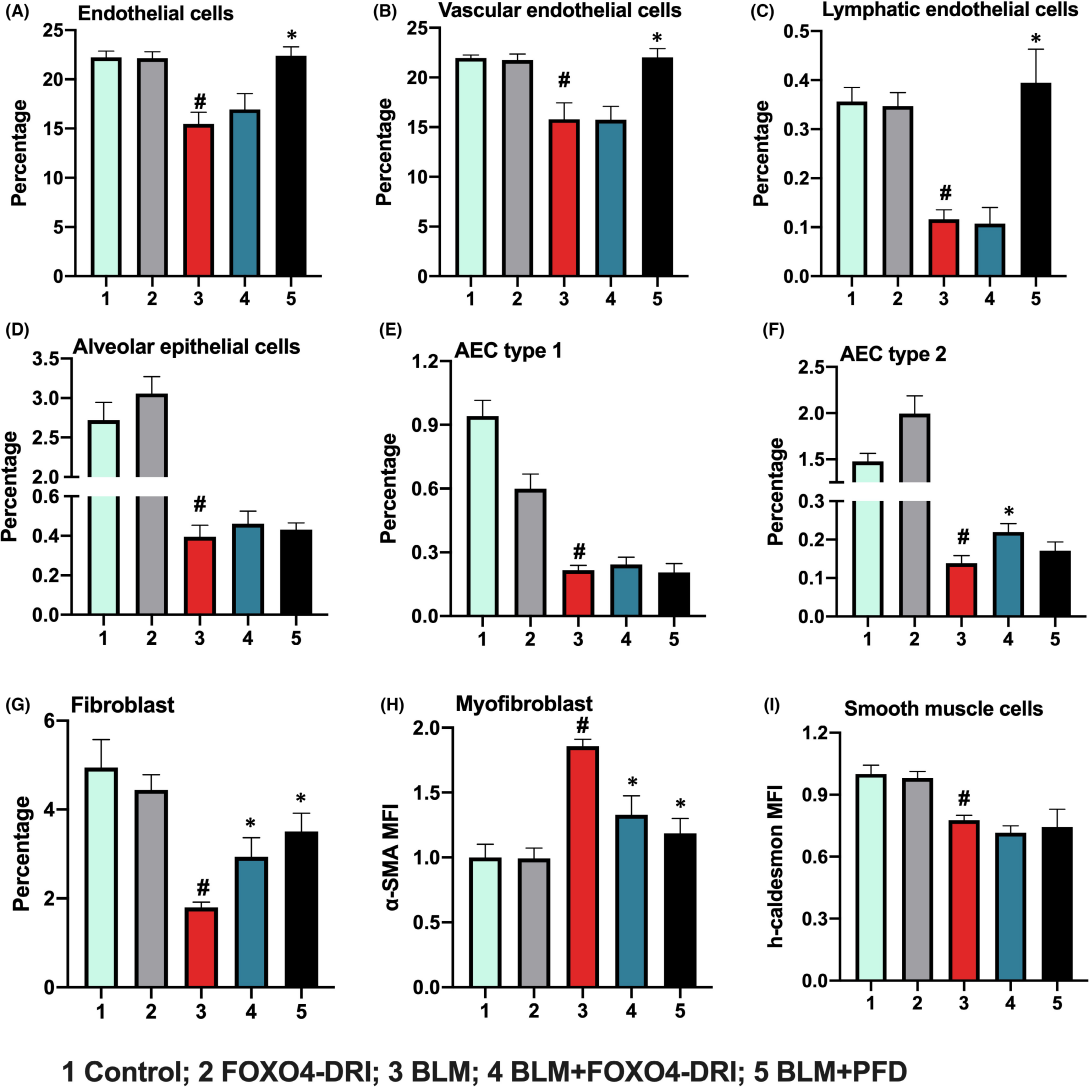
FOXO4‐DRI improves the impaired ratio of lung cells. The BLM‐induced PF model were established and mice were treated with FOXO4‐DRI or PBS, then lung cells were obtained as described in Materials and Methods. Bar Graphs show the percentage of (A) Endothelial cells; (B) Vascular endothelial cells; (C) Lymphocytic endothelial cells; (D) Alveolar epithelial cells(AECs); (E) Type 1 alveolar epithelial cells (AEC type1); (F) Type 2 alveolar epithelial cells (AEC type2); (G) Fibroblasts; Mean fluorescence intensity (MFI) of (H) Myofibroblasts and (I) Smooth muscle cells. All the data were represented as mean ± SEM (*n* = 5 in panel A–I). ^#^ *p *< 0.05 vs control; *p < 0.05 vs BLM

In the lung injury‐repair, activation of fibroblast‐to‐myofibroblasts differentiation is one of the most important factor to deposit ECM and support the regeneration of type 2 AECs.^24^ The lung is composed of many cell types, including endothelial cells, AECs, fibroblasts and myofibroblasts; endothelial cells can be further sorted as vascular endothelial cells and lymphocytic endothelial cells; AECs can be sorted as AEC 1 and AEC 2.^13^ To further explore the effect of FOXO4‐DRI on BLM‐induced cell injury in lung, we detected the changes of the above cells in lung using a flow cytometer. As shown in Figure 2 and Figure S2, compared with control group, there was significant decrease in the percentage of endothelial cells, AECs, fibroblast, MFI of smooth muscle cells and increase in MFI of myofibroblasts after mice were treated with BLM; compared to BLM group, FOXO4‐DRI could elevated the percentage of AEC2 and fibroblast and reduced the MFI of myofibroblasts. Notably, PFD could not increase the cell number of AEC2 in our experiment, also, FOXO4‐DRI could not improve the percentage of endothelial cells, which can be rescued by PFD; Above data suggested that FOXO4‐DRI may rescue BLM‐induced PF through eliminate myofibroblasts and promote recovery of fibroblast and AEC2 number.

### 3.3 FOXO4‐DRI works on the extracellular matrix (ECM) receptor interaction pathway to mitigate BLM‐induced PF

**FIGURE 3:**
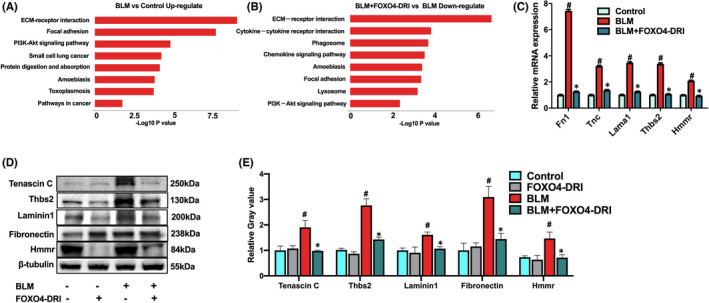
FOXO4‐DRI downregulates expression of proteins in ECM (extracellular matrix) receptor interaction pathway to mitigate BLM‐induced PF. RNA‐Seq and Kyoto Encyclopedia of Genes and Genomes (KEGG) pathway enrichment analysis was performed using lung tissues from control, BLM and BLM+FOXO4‐DRI groups. (A) The upregulated pathways BLM vs control; and (B) the downregulated pathways BLM+FOXO4‐DRI vs BLM; (C) The relative expression of Fn1, Tnc, Lama1, Thbs2 and Hmmr mRNA in the lung tissue; (D) Representative Western blot images of Fn1, Tnc, Lama1, Thbs2 and Hmmr in the lung tissues. (E) Relative grey value of protein expression. All the data were represented as mean ± SEM (*n* = 5 in panel C, *n* = 3 in panel E). ^#^ *p *< 0.05 vs control; *p < 0.05 vs BLM

FOXO4‐DRI ameliorated BLM‐induced PF and did not affect the histological morphology of normal lung. To further decipher the protective mechanism of FOXO4‐DRI, we performed RNA‐Seq and Kyoto Encyclopedia of Genes and Genomes (KEGG) pathway enrichment analysis using lung tissues from control, BLM and BLM+FOXO4‐DRI groups. The KEGG pathway enrichment analysis revealed that, compare with control, the significant elevated top genes were mostly involved in ECM‐receptor interaction, focal adhesion and PI3K‐Akt signalling pathway in BLM group. Compare with those in BLM group, the top genes exhibiting significantly decreased expression were mostly involved in ECM‐receptor interaction, cytokine‐cytokine receptor interaction, phagosome, chemokine signalling pathway, focal adhesion and PI3K‐Akt signalling pathway. (Figure 3A,B). There were 24 overlap genes involved in in all the control, BLM and BLM+FOXO4‐DRI group (Figure S3A). We performed quantitative real‐time PCR in these overlap genes to verify the results of RNA‐Seq analysis. The protein level of ECM**‐**receptor interaction pathway, including Fn1, Tnc, Lama1, Thbs2 and Hmmr was consistently with that in mRNA level, and FOXO4‐DRI downregulated these expressions in both gene and protein level (Figure 3C–E). These results indicated that FOXO4‐DRI worked on ECM‐receptor interaction pathway in mice to ameliorate BLM‐induce PF.

Among these overlap genes, BLM downregulated the expression of Sele, Cytl1, Dpysl5, Gc, Nrgn genes, which are mainly involved in cell adhesion,^25^ inflammatory response,^26^ nerve conduction,^27^ signal transduction,^28^ material transport and metabolism,^29^, ^30^ and FOXO4‐DRI attenuated the depression of these gene expressions (Figure S3B). BLM also upregulated the expression of Chil4, Pappa2, Gpr176, Krt6a, Mex3a, Ntrk2, Tgm5 and Myh2 genes, which are mainly involved in regulations of signal transduction,^31^, ^32^, ^33^ material metabolism^34^, ^35^, ^36^ and cell junctions.^37^ FOXO4‐DRI downregulated BLM‐induced expressions of Chil4, Pappa2, Gpr176, Krt6a, Mex3a, Ntrk2, Tgm5 to nearly normal levels and elevated the expression of Myh2, a gene associated with tight junction.^37^ (Figure S3C).

### 3.4 FOXO4‐DRI alleviates myofibroblast differentiation in BLM‐induced PF mouse

**FIGURE 4:**
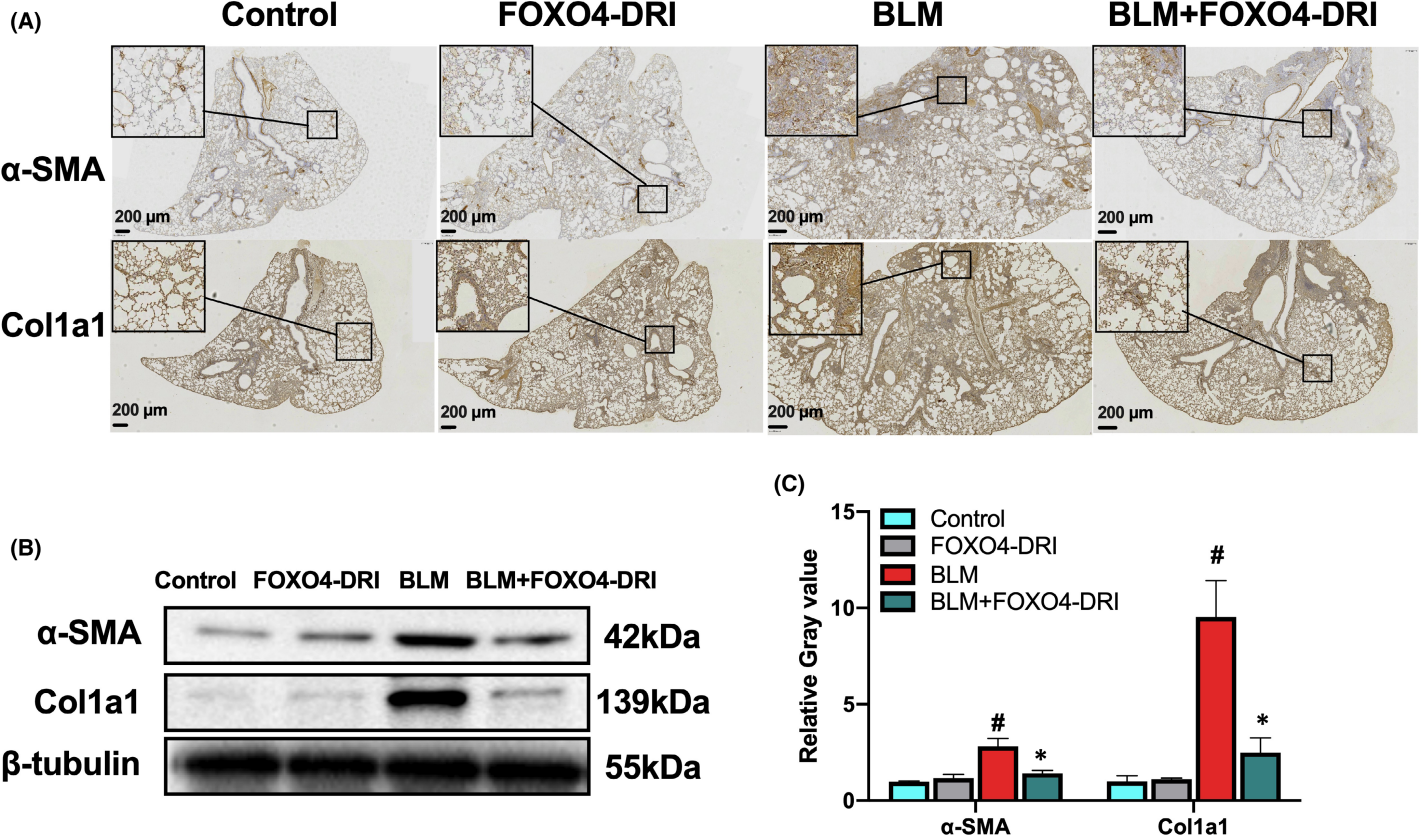
FOXO4‐DRI alleviates myofibroblast differentiation in BLM‐induced PF mouse. IHC experiments were executed using lung tissues as described in Materials and Methods. (A) Representative scanned IHC images of α‐SMA and Col1a1 in lung tissues; (B) Representative WB images of α‐SMA and Col1a1 in lung tissues; (C) Relative grey value of protein expression. All the data were represented as mean ± SEM (*n* = 3 in panel C). ^#^ *p *< 0.05 vs control; *p < 0.05 vs BLM

Alpha‐SMA is used as the myofibroblast marker according to previously reports,^38^, ^39^ Col1a1 is one of the components of ECM, which upregulated in TGF‐β‐induced myofibroblast differentiation,^40^, ^41^ so we detected the expression of both two proteins to further confirm the effect of FOXO4‐DRI on BLM‐induced myofibroblasts. As shown in Figure 4A, IHC images showed that there was significant increase of α‐SMA and Col1a1 positive staining in BLM‐induced mice lung section, FOXO4‐DRI inhibited the upregulation of α‐SMA and Col1a1 proteins. WB results showed a consistently effect of FOXO4‐DRI on BLM‐induced upregulation of α‐SMA and Col1a1 (Figure 4B,C). Above data indicating that FOXO4‐DRI could eliminate BLM‐induced myofibroblast differentiation.

### 3.5 FOXO4‐DRI alleviates TGF‐β‐induced myofibroblast differentiation *in vitro*

**FIGURE 5:**
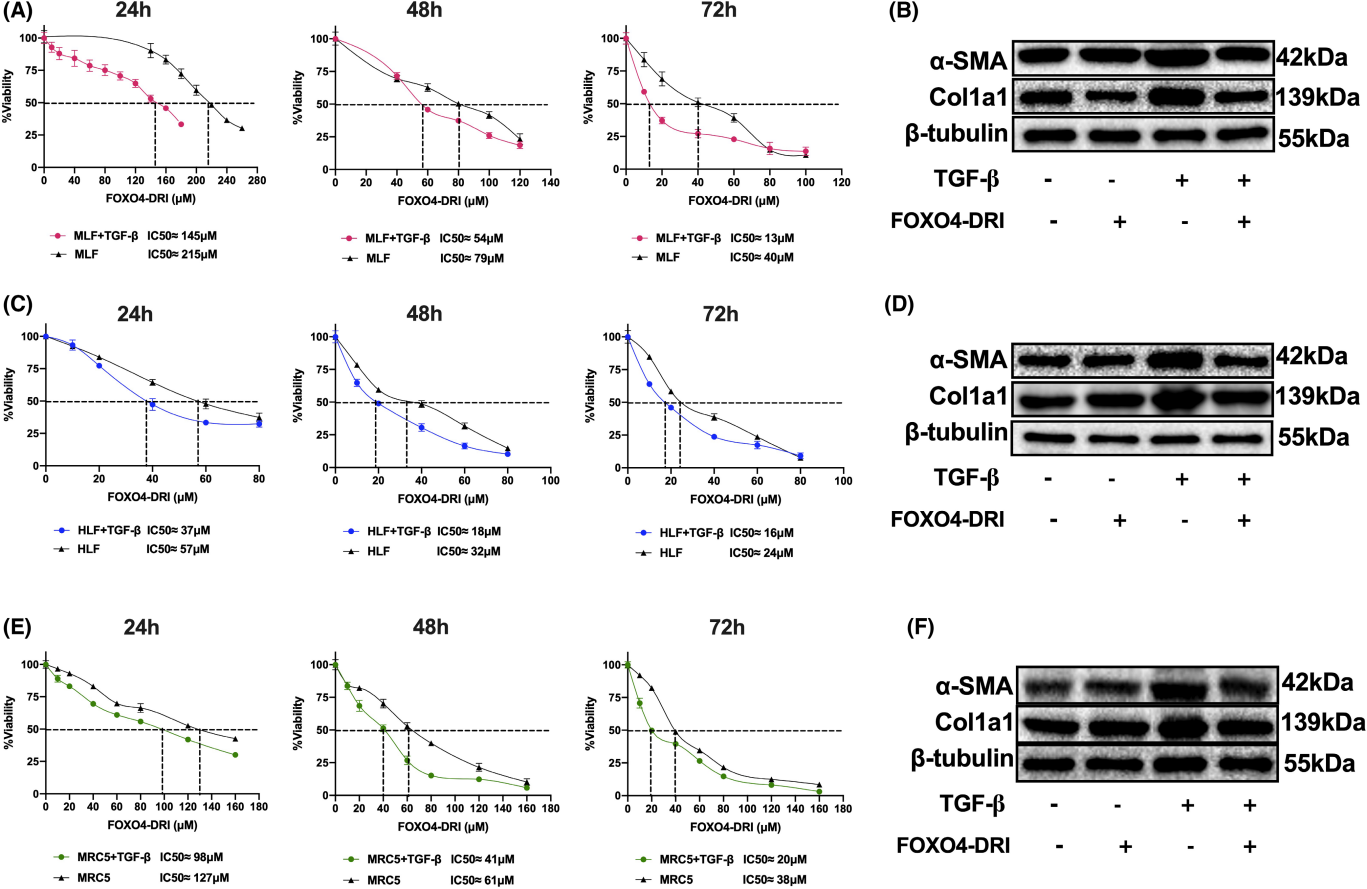
FOXO4‐DRI is inclined to kill TGF‐β‐induced myofibroblasts in vitro MLF, HLF and MRC5 cells were co‐cultured with TGF‐β and FOXO4‐DRI as described in Materials and Methods. (A) IC50 value of FOXO4‐DRI on MLF and TGF‐β‐treated MLF at 24, 48 and 72 h; (B) Representative WB images of α‐SMA and Col1a1 in MLF; (C) IC50 value of FOXO4‐DRI on HLF and TGF‐β‐treated HLF at 24, 48 and 72 h; (D) Representative WB images of α‐SMA and Col1a1 in HLF; (E) IC50 value of FOXO4‐DRI on MRC5 and TGF‐β‐treated MRC5 at 24, 48 and 72 h; (F) Representative WB images of α‐SMA and Col1a1 in MRC5; All the data were represented as mean ± SEM (*n* = 5 in panel A, C and E)

TGF‐β is one of the major pro‐fibrotic cytokines for myofibroblast differentiation, it was always used to stimulate the fibroblast‐to‐myofibroblast differentiation in vitro.^38^, ^40^, ^42^ In this study, we use 4ng/mL TGF‐β to stimulate MLF, HLF and MRC5 cell line for 24 h, then different concentration of FOXO4‐DRI was co‐cultured with cells for 24, 48 and 72 h. As shown in Figure 5, the half maximal inhibitory concentration (IC50) value of FOXO4‐DRI on MLF was 215, 79 and 40 μM, respectively, IC50 value of FOXO4‐DRI on TGF‐β‐stimulated MLF was 145, 54 and 13 μM, respectively, at the time point of 24, 48 and 72 h (Figure 5A); IC50 value of FOXO4‐DRI on HLF was 57, 32, 24, 37, 18, and 16 μM, respectively, at the time point of 24, 48 and 72 h (Figure 5C); IC50 value of FOXO4‐DRI on MRC5 was 127, 61, 38, 98, 41, and 20 μM, respectively, at the time point of 24, 48 and 72 h (Figure 5E). There were 1.3–3 times IC50 value of FOXO4‐DRI on lung fibroblast compared to the TGF‐β‐stimulated group. WB results of α‐SMA and Col1a1confirmed the rescue of FOXO4‐DRI on myofibroblast (Figure 5B, D and F). Above data indicated that FOXO4‐DRI is more inclined to kill myofibroblast.

### 3.6 FOXO4‐DRI induces more apoptotic cells in TGF‐β‐induced myofibroblasts in vitro

**FIGURE 6:**
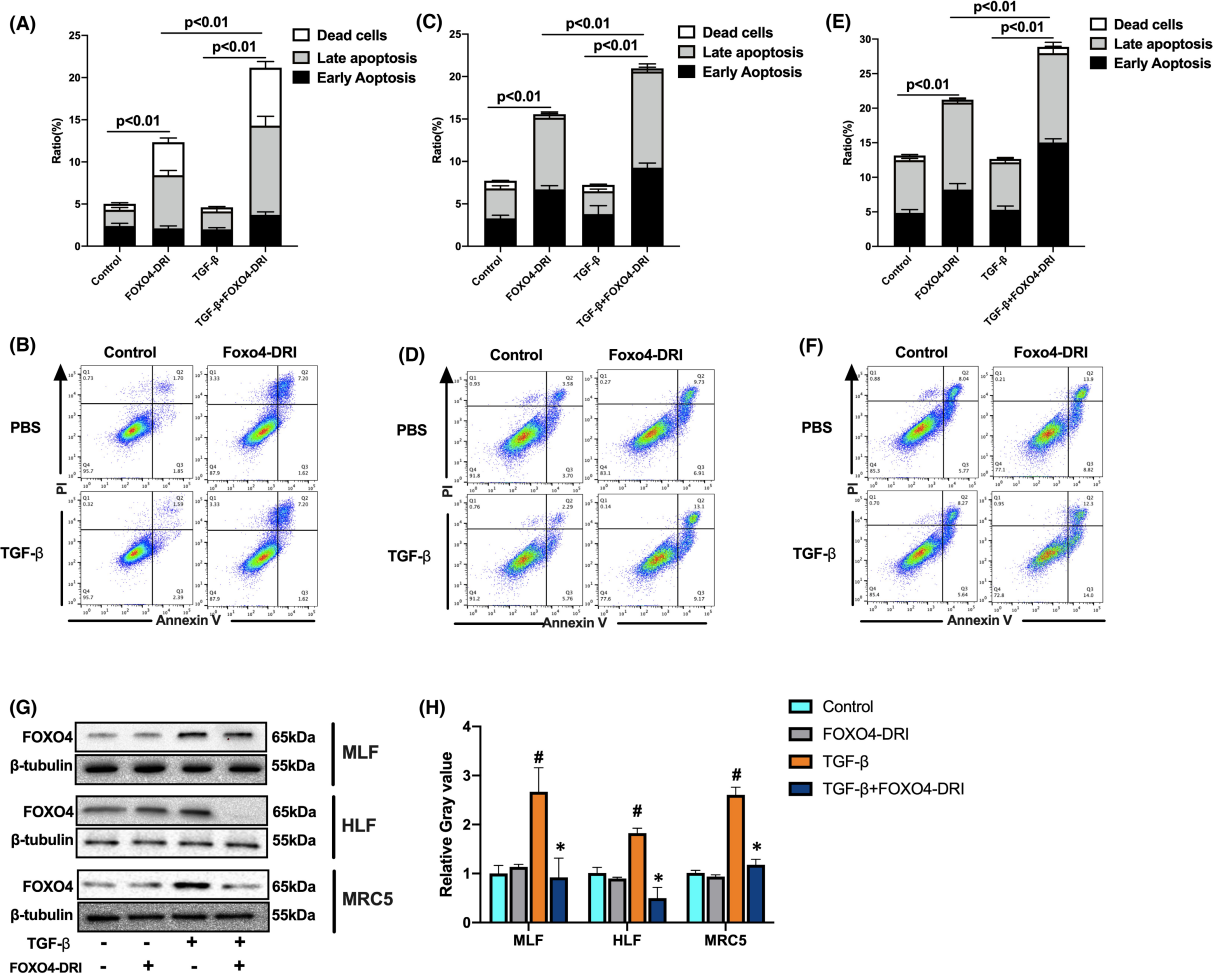
FOXO4‐DRI induces more apoptotic cells in TGF‐β‐induced myofibroblasts in vitro MLF, HLF and MRC5 cells were co‐cultured with TGF‐β for 24 h and then treated with FOXO4‐DRI for 4 h as described in Materials and Methods. (A) Ratio of apoptotic and dead cells in MLF; (B) Representative FACS scatters show the gating strategy; (C) Ratio of apoptotic and dead cells in HLF; (D) Representative FACS scatters show the gating strategy; (E) Ratio of apoptotic and dead cells in MRC5; (F) Representative FACS scatters show the gating strategy; (G) Representative WB images of FOXO4 in MLF, HLF and MRC5; (H) Relative grey value of FOXO4 protein expression. All the data were represented as mean ± SEM (*n* = 5 in panel A, C, E and *n* = 3 in panel H)

To explore the underlying mechanisms that FOXO4‐DRI kills myofibroblast, we detected cell apoptosis in MLF, HLF and MRC5. As shown in Figure 6A–F, after cells were co‐cultured with FOXO4‐DRI, the apoptotic and dead cells increase 7.3% (MLF), 7.7% (HLF) and 6.4% (MRC5) compare with control groups. TGF‐β treatment did not increase cell apoptosis; after cells were co‐cultured with FOXO4‐DRI, the apoptotic and dead cells increase 8.9% (MLF), 5.5% (HLF)and 8.0% (MRC5) compare with TGF‐β groups, suggesting a tendency of FOXO4 induced myofibroblast apoptosis. In senescent cells, there is upregulation of FOXO4 expression, FOXO4‐DRI perturbs interaction between FOXO4 and p53 and induces cell apoptosis. So the protein level of FOXO4 was detected in our experiment, as shown in Figure 6G,H, FOXO4 protein was upregulated after treatment with TGF‐β, FOXO4‐DRI reverse the protein expression, which should be attribute to the clearance of FOXO4‐riched cells. Above data suggests that FOXO4‐DRI is more inclined to kill myofibroblast by inducing cell apoptosis.

## 4 DISCUSSION

PF has received attention because of its poor prognosis and its unresponsiveness to traditional therapies.^23^ In recent years, the novel strategy of targeting senescent cells to treat PF has been supported by some researches.^6^, ^43^, ^44^ Hashimoto and his colleagues provide crucial evidence that cell senescence contributes to lung ageing, and elimination of naturally occurring senescent cells promote the recovery of lung structure and function in aged mice.^9^ Schafer and his colleagues reported that senescent cell ablation attenuates BLM‐induced impairment in lung function.^6^ Pan and his colleagues have reported that ABT‐263, a senolytic agent, selectively kills senescent cells and reserves radiation‐induced PF in mouse.^18^ Cumulatively, the strategy of senescent cell elimination exhibits a therapeutic potential on PF.

In this study, we treated the BLM‐induced PF mouse model with FOXO4‐DRI, and found that besides the elimination of senescent cells and SASP, FOXO4‐DRI could attenuate BLM‐induced morphological changes and collagen deposition to the similar as the approved medication PFD. Furthermore, FOXO4‐DRI increases the percentage of type 2 alveolar epithelial cells (AEC2) and fibroblasts and decreases the myofibroblasts in BLM‐induced PF mouse models. Compared to mouse and human lung fibroblast cell lines, FOXO4‐DRI is inclined to kill TGF‐β‐induced myofibroblast in vitro. The inhibited effect of FOXO4‐DRI on myofibroblast and ECM proteins lead to a downregulation of ECM‐receptor interaction pathway in BLM‐induced PF. Above all, FOXO4‐DRI ameliorates BLM‐induced PF in mouse and may be served as a viable therapeutic option for PF.

Activation of fibroblast‐to‐myofibroblasts differentiation is one of the most important factor to deposit ECM.^24^ In this study, FOXO4‐DRI is more inclined to kill the myofibroblasts both in vivo and in vitro. The upregulation expression of FOXO4 protein in TGF‐β‐stimulated cells may provide the possible reason for the effect of FOXO4‐DRI. The formation of ECM also requires the secretion of ECM proteins. ECM is achieved by following a strict layered assembly pattern, which begins with the deposition of fibronectin filaments on the cell surface, a process called fibrillary formation.^45^ In this study, FOXO4‐DRI downregulated the main ECM protein significantly, which mainly contributed for the reducing of PF degree. Therefore, we summarize the whole process of FOXO4‐DRI works on BLM‐induced PF as follows, FOXO4‐DRI eliminated senescent cells, downregulation of SASP released by senescent cells reduce the inflammatory stimulation to neighbour cells to rescue BLM‐induced PF; On the other hand, FOXO4‐DRI is more inclined to kill myofibroblasts, downregulated the expression of main ECM proteins, reduced ECM formation and finally inhibited ECM‐receptor interaction to mitigate BLM‐induced PF.

PFD has been approved in Europe in 2011 for the treatment of IPF and in the USA in 2014.^46^ The recommended daily maintenance dose of PFD is 801 mg three times per day (2403 mg/day) with a 14‐day titration.^46^ Consistently with our results, it has been reported that PFD could ameliorate BLM‐Induced PF at administration of 300 mg/kg PFD for 3–4 weeks immediately after BLM treatment.^47^ Obviously, PFD needs to be given at very early time point in mouse, and the medication period is long. Compared with PFD, BLM‐induced lung fibrosis mouse was treated with 5 mg/kg FOXO4‐DRI for 3 times, started from 14^th^ day after BLM treatment; so the advantage over PFD, FOXO4‐DRI lies in its short administration time and the low treatment dose. Importantly, the same as other senolytic agents, it can be used to treat PF even when pathological changes have occurred. FOXO4‐DRI is a cell‐penetrating peptide, which can in theory target any surface‐exposed stretch of amino acids to block specific protein‐protein interactions, selectively modulate specific signalling events.^48^ Thus it have the advantages of target specificity and low toxicity over the reported senolytic agents.^11^ In addition, in the treatment of PF, FOXO4‐DRI may reduce the risk of cancer progression and metastasis by eliminating senescent cells, which have been demonstrated to contribute to cancer metastasis and relapse.^49^, ^50^ At present, mouse was administrated with FOXO4‐DRI by intraperitoneal injection, it will be better to be improved as an oral gavage peptide. Above all, FOXO4‐DRI has great potential as a candidate for PF therapy.

## CONFLICT OF INTEREST

The authors declare no conflicts of interest.

## AUTHOR CONTRIBUTIONS

**Xiaodan Han:** Data curation (equal); Methodology (equal); Project administration (equal); Resources (equal); Software (equal); Writing – original draft (equal). **Tong Yuan:** Formal analysis (equal); Methodology (equal); Project administration (equal); Software (equal); Validation (equal); Writing – original draft (equal). **junling zhang:** Conceptualization (equal); Supervision (equal); Writing – review & editing (equal). **Yonggang Shi:** Methodology (supporting); Supervision (supporting). **Deguan Li:** Methodology (supporting); Supervision (supporting). **yinping dong:** Methodology (supporting); Supervision (supporting). **saijun Fan:** Methodology (equal); Project administration (equal); Supervision (equal); Writing – review & editing (equal).

## Supporting information

Fig S1

Fig S2

Fig S3

Fig S4

## Data Availability

Data openly available in a public repository that issues datasets with DOIs.
