# Correction to “FOXO4 peptide targets myofibroblast ameliorates bleomycin‐induced pulmonary fibrosis in mice through ECM‐receptor interaction pathway”Han XD, Yuan T, Zhang JL, et al. FOXO4 peptide targets myofibroblast ameliorates bleomycin‐induced pulmonary fibrosis in mice through ECM‐receptor interaction pathway. J Cell Mol Med. 2022;26(11):3269‐3280

**DOI:** 10.1111/jcmm.18502

**Published:** 2024-08-21

The author observed errors in Figure 6B. The representative FACS scatters in the lower right corner were duplicated with that in the upper right corner. After checking the original data, authors found that the FACS scatters in the PBS+ Foxo4‐DRI group were misplaced in TGF‐β + Foxo4‐DRI group (lower right corner). All the authors sincerely apologize for any confusion this may have caused and confirm that the scientific conclusion of the article remains unchanged.

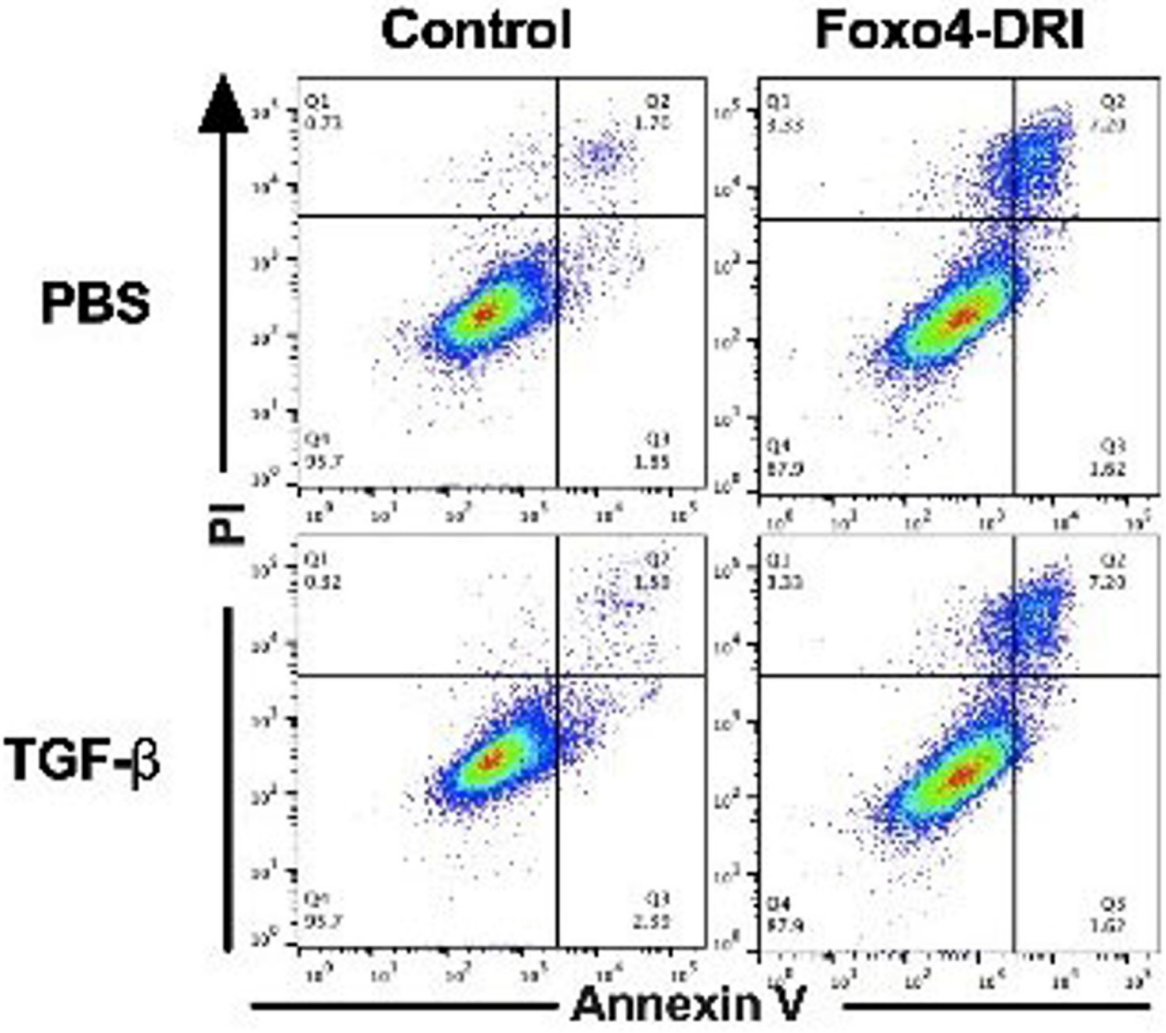

**Corrected Figure 6B**

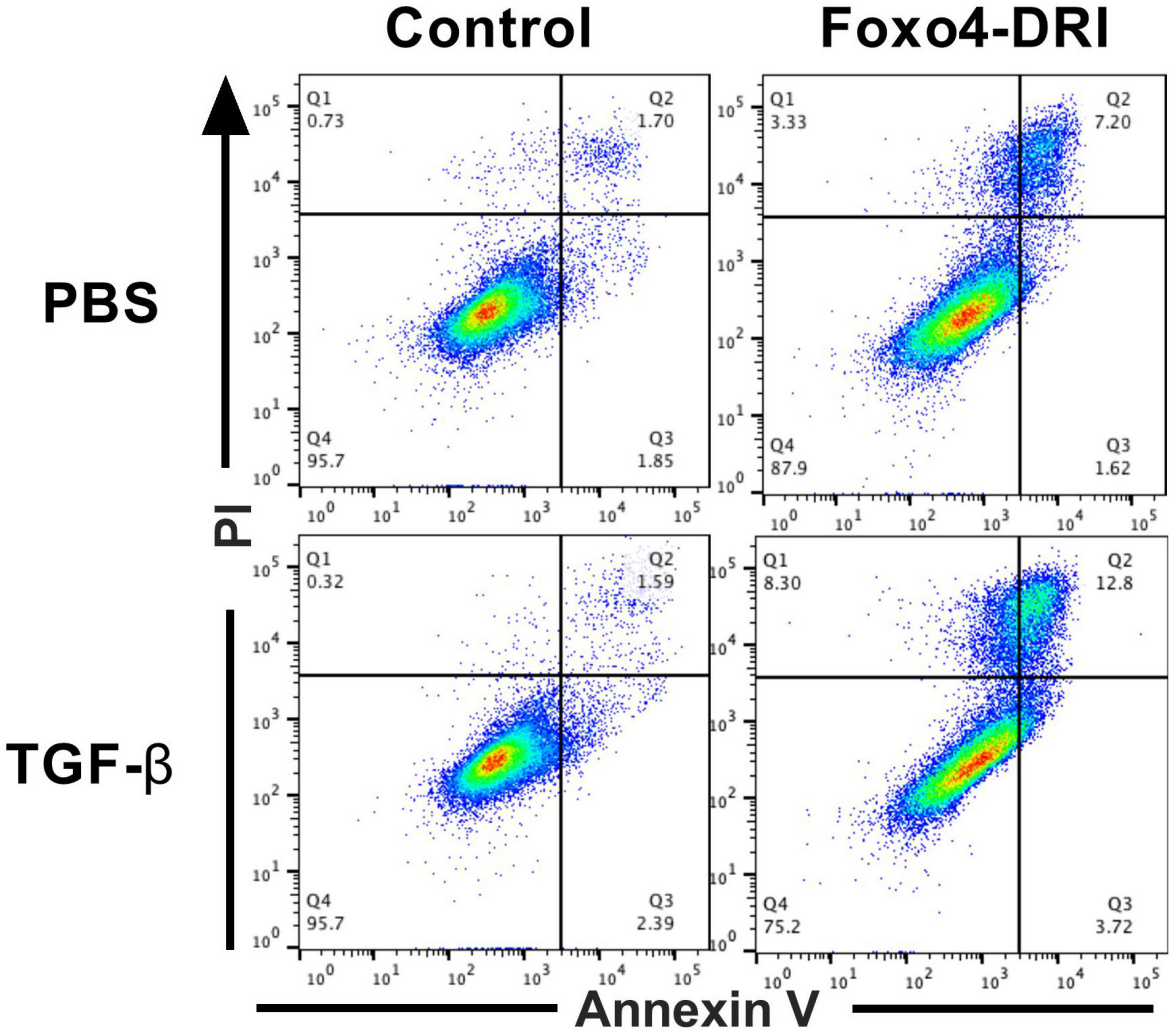

FIGURE 6. FOXO4‐DRI induces more apoptotic cells in TGF‐β‐induced myofibroblasts in vitro MLF, HLF and MRC5 cells were co‐cultured with TGF‐β for 24 h and then treated with FOXO4‐DRI for 4 h as described in Materials and Methods. (A) Ratio of apoptotic and dead cells in MLF; (B) Representative FACS scatters show the gating strategy; (C) Ratio of apoptotic and dead cells in HLF; (D) Representative FACS scatters show the gating strategy; (E) Ratio of apoptotic and dead cells in MRC5; (F) Representative FACS scatters show the gating strategy; (G) Representative WB images of FOXO4 in MLF, HLF and MRC5; (H) Relative grey value of FOXO4 protein expression. All the data were represented as mean ± SEM (*n* = 5 in panel A, C, E and *n* = 3 in panel H).
