# Supplementary figures and images for: FOXO4 peptide targets myofibroblast ameliorates bleomycin‐induced pulmonary fibrosis in mice through ECM‐receptor interaction pathway
Source: J Cell Mol Med. 2022 May 5;26(11):3269–80. doi: 10.1111/jcmm.17333 (PMC9170815; doi:10.1111/jcmm.17333)

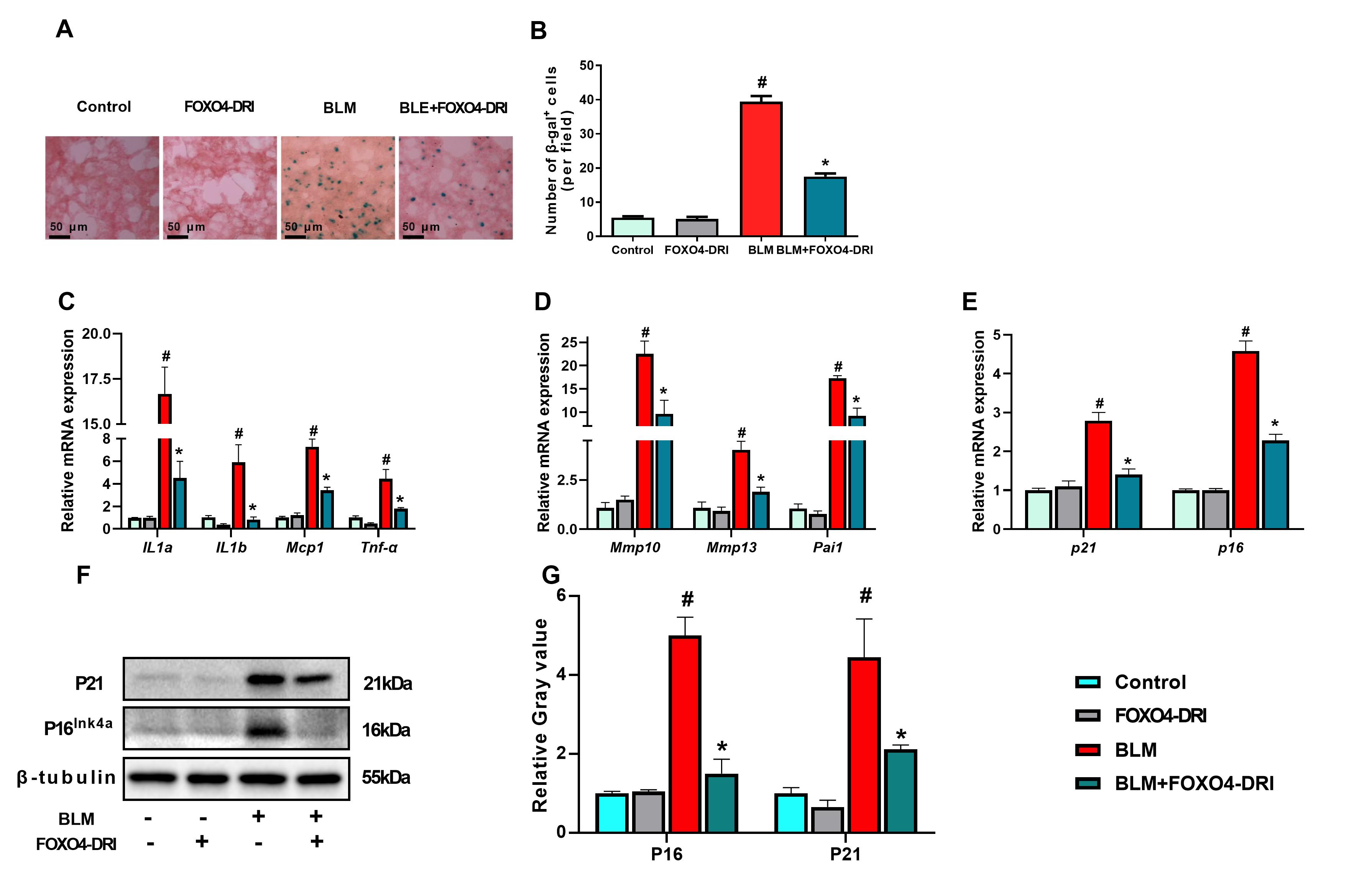

Supplement: Supplementary file 1 — Fig S1 [file JCMM-26-3269-s001.jpg]

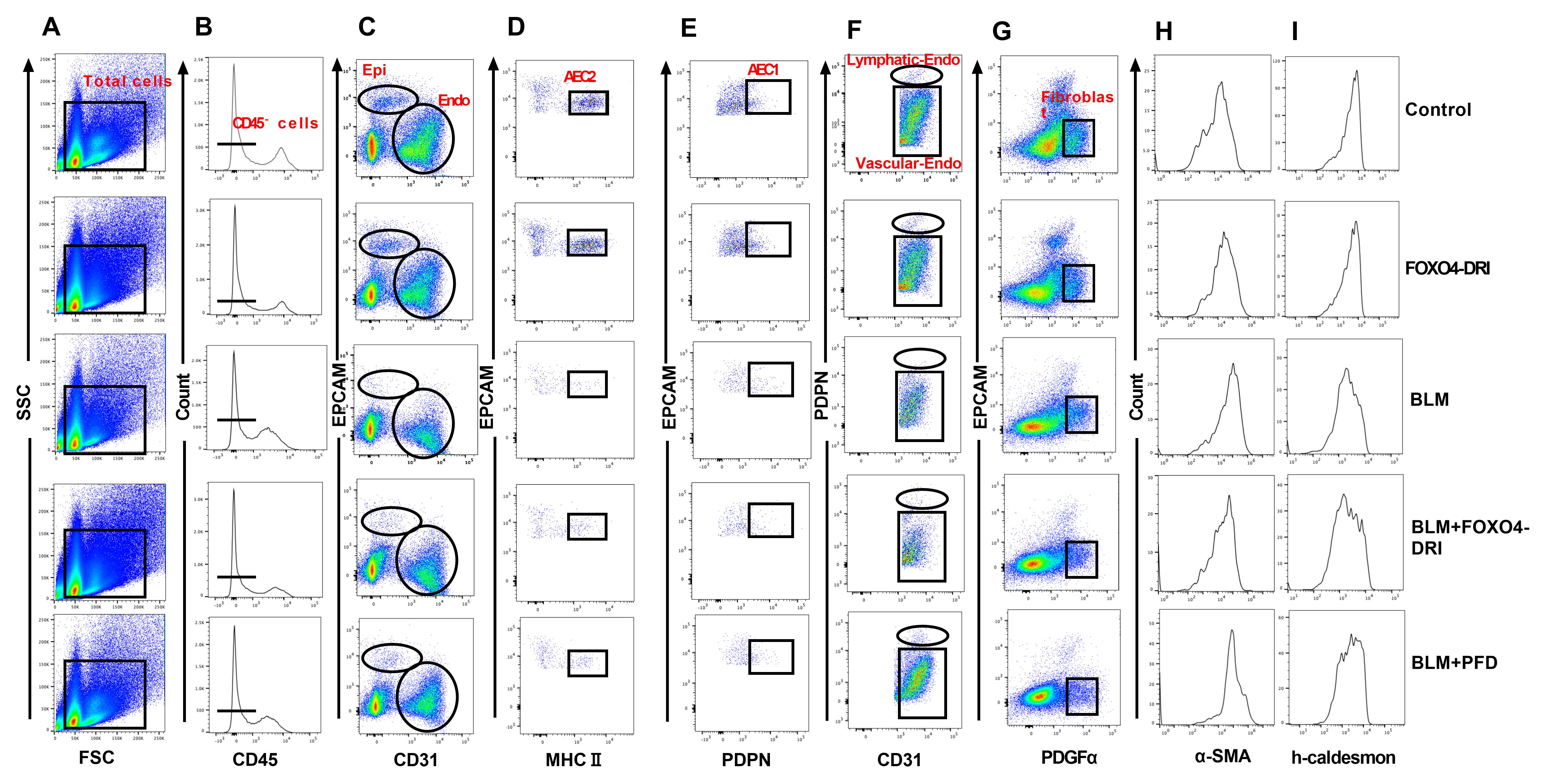

Supplement: Supplementary file 2 — Fig S2 [file JCMM-26-3269-s003.jpg]

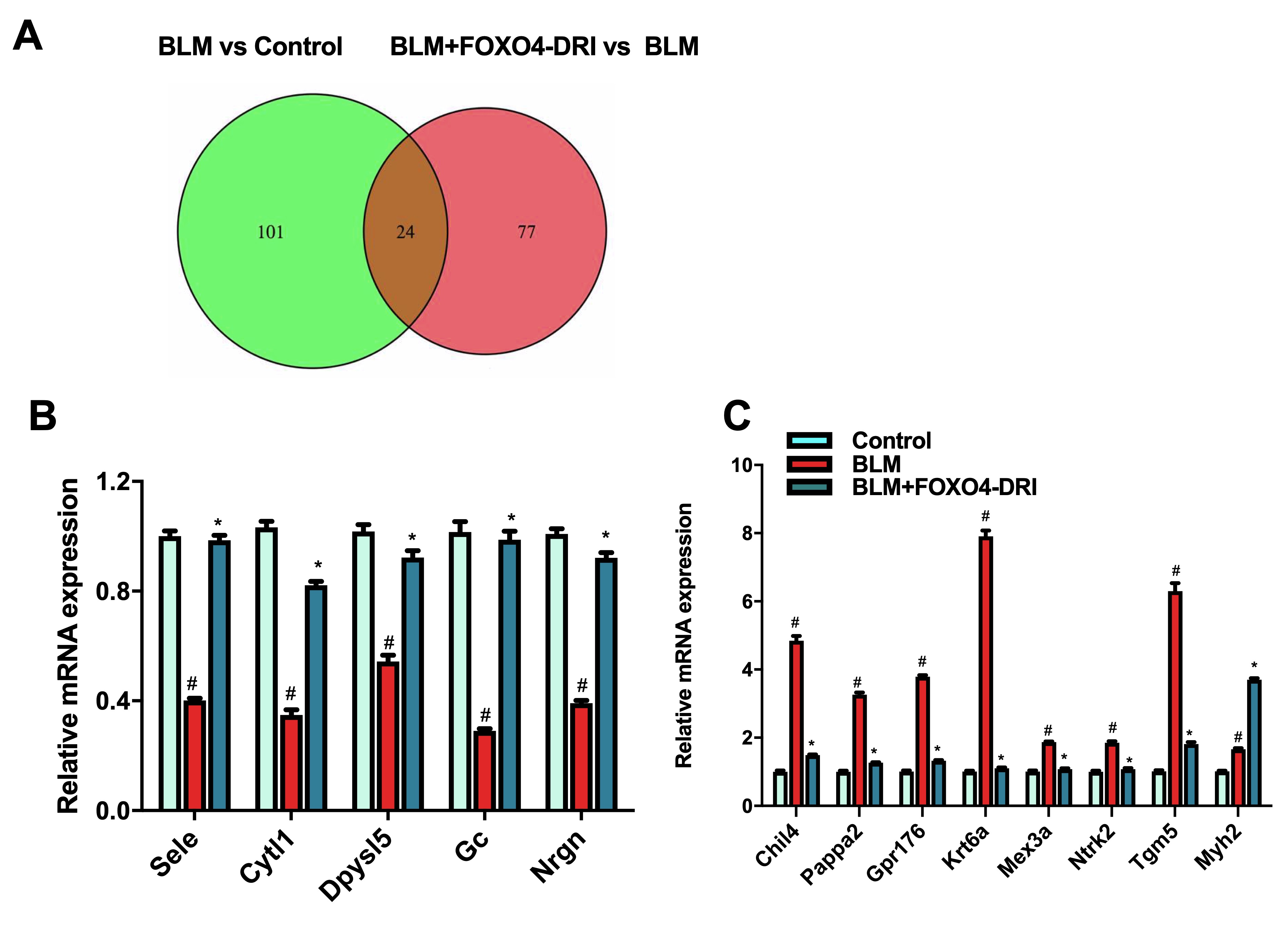

Supplement: Supplementary file 3 — Fig S3 [file JCMM-26-3269-s002.jpg]

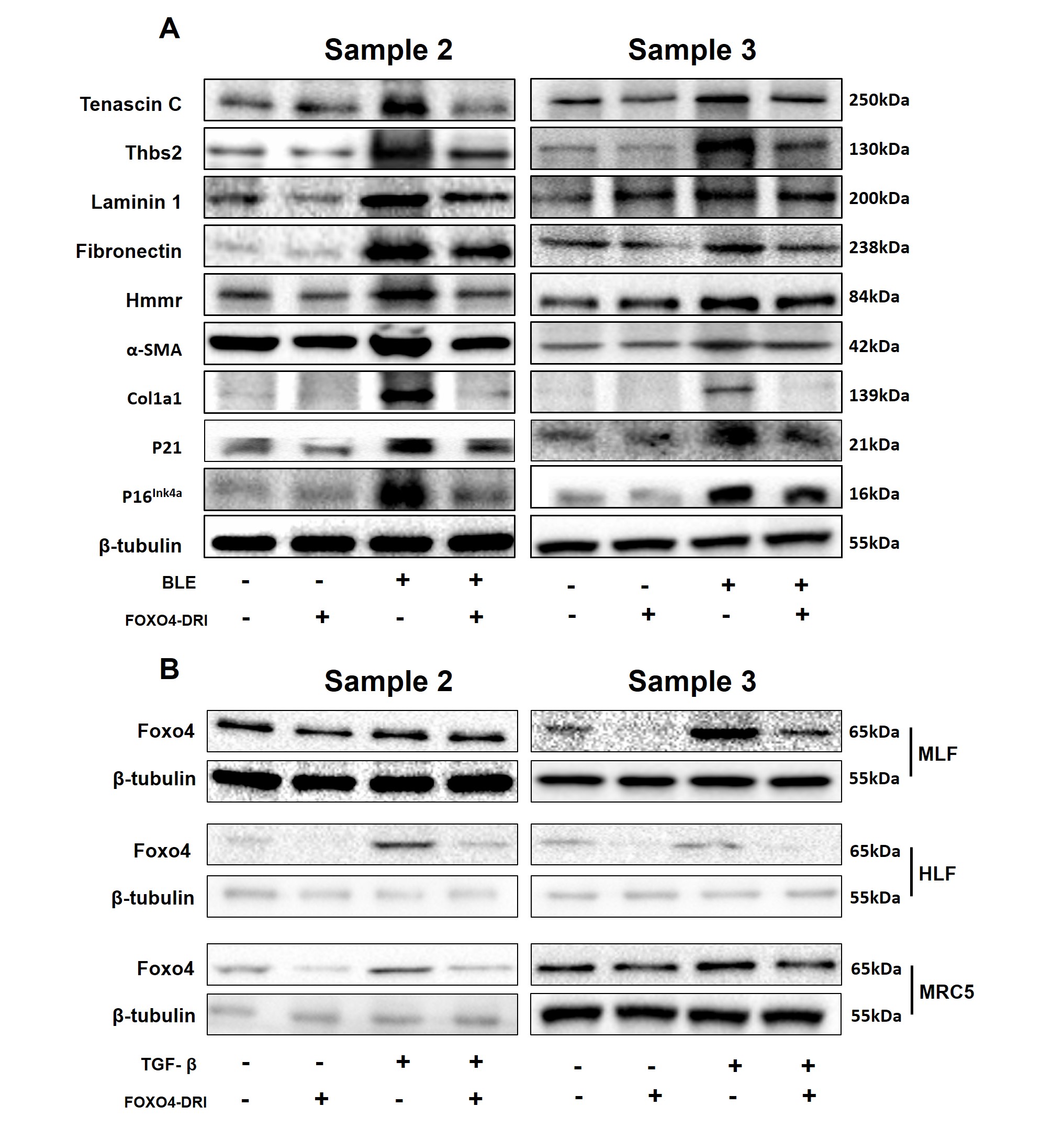

Supplement: Supplementary file 4 — Fig S4 [file JCMM-26-3269-s004.jpg]
